# Effects of *Cnidium officinale*, *Pueraria lobata* Ohwi, and *Leonurus japonicus* Extract on Vascular Endothelial Dysfunctions in Ovariectomized Rats and Molecular Mechanisms

**DOI:** 10.3390/ijms26104708

**Published:** 2025-05-14

**Authors:** Joohee Oh, Minseo Kim, Jinsoo Kim, Jiwon Jang, Dongjin Noh, Hyun-Sook Kim

**Affiliations:** 1 Department of Food and Nutrition, College of Human Ecology, Sookmyung Women’s University, Seoul 04310, Republic of Korea; 2 Natural Products Convergence R&D Division, Kwangdong Pharm Co., Ltd., Gwacheon 13840, Republic of Korea

**Keywords:** menopause, cardiovascular diseases, ovariectomized rats, vascular endothelial dysfunction

## Abstract

Menopause is the natural period of aging in women induced by ovary deterioration, resulting in estrogen deficiency. We evaluated the antioxidative and anti-inflammatory properties of *Cnidium officinale*, *Pueraria lobata* Ohwi, and *Leonurus japonicus* (CPL) extracts on vascular endothelial dysfunction. After treatment, CPL extracts decreased serum lipid profiles, serum vasoactive substances, tail temperatures, and cardiovascular risk indices. In ovariectomized rats, vasodilation significantly increased, with an increase in endothelial nitric oxide synthase (eNOS) in the CPL200 and CPL500 groups compared with the OVX group (*p* < 0.05). The extracts also significantly reduced vascular cell adhesion protein 1 (VCAM-1) in the CPL50, CPL100, and CPL200 groups compared with the OVX group (*p* < 0.05, *p* < 0.01, and *p* < 0.001, respectively). Intercellular adhesion molecule 1 (ICAM-1) was also reduced in the CPL100 and CPL200 groups compared with the OVX group (*p* < 0.001 and *p* < 0.0001, respectively); this was achieved through the downregulation of the nuclear factor kappa-light-chain-enhancer of activated B cells (*NF-κB*) and inducible nitric oxide (*iNOS*), which resulted in the synthesis of nuclear factor erythroid 2-related factor 2 (*NRF2*) and *eNOS* in HUVECs. Our results show that CPL extracts could provide cardioprotective effects against vascular endothelium dysfunction by decreasing inflammation and upregulating vasodilation, ascertained by evaluating the antioxidant systems of ovariectomized rats. Further studies are needed to explore the long-term cardioprotective effects.

## 1. Introduction

Menopause, which most women experience in their late 40s or early 50s, is a normal aspect of aging. It is characterized by a decline in ovarian follicular activity [1]. During menopause, hormone levels (including estrogen) begin to decrease in women, driving an increase in weight and even cardiovascular diseases [2]. Due to its potential cardioprotective effects, estrogen may reduce the risk of certain chronic diseases, including cardiovascular disorders and osteoporosis. The relationship between estrogen and cancer risk remains controversial, and cardioprotective effects in postmenopausal women or those undergoing hormone replacement therapy have not been fully established [3].

Hormone replacement therapy (HRT) is the primary treatment for menopausal symptoms. However, HRT has certain adverse effects, including weight gain and an increased risk of ovarian, endometrial, and breast cancers [4]. Therefore, it is necessary to develop alternative medicines that have no adverse effects.

*Cnidium officinale* (*C. officinale*), is also known by the synonym *Conioselinum officinale* (Makino) K.Ohashi & H.Ohashi; *Pueraria lobata* Ohwi (*P. lobata* Ohwi), is also known by the syonym *Pueraria montana var. lobata* (Willd.) Maesen & S.M.Almeida ex Sanjappa & Predeep; and *Leonurus japonicus* Houtt (*L. japonicus*). are traditional medicines widely used to treat diseases in women due to their antioxidative and anti-inflammatory properties [5]. *C. officinale*, the dried root stem of *C. officinale* Makino, has natural antioxidant properties that can be used to treat menstrual irregularities, inflammation, and blood pressure [6]. *C. officinale* has significant components of falcarindiol, 6-hydroxy-7methoxydihydroligustilide, and ligusilidiol, which demonstrate anti-inflammatory activity [7]. *P. lobata* Ohwi roots are used in traditional Chinese medicine therapy to lower the body temperature, treat hypertension, enhance blood flow, and lower the risk of heart disease [8]. *P. lobata* Ohwi contains isoflavones such as puerarin, daidzin, and daidzein, which have vasodilator, antioxidant, and anti-inflammatory properties [9]. *L. japonicus* can enhance blood circulation by removing blood stasis, dissipating the heat produced by internal inflammation. Consequently, it effectively treats irregular or painful menstruation [10]. *L. japonicus* is rich in alkaloids, which have proven beneficial effects in cardiocerebrovascular disease, and leonurine, which produces antioxidant and anti-inflammatory effects [11].

Menopause—a process intimately linked to the aging process—is one of the most important developmental milestones in a woman’s life. Further studies into this process are necessary, as its physiological implications on the dysfunction of the vascular endothelium remain unclear.

The aim of this study was to evaluate the cardioprotective effects of mixtures comprising *C. officinale*, *P. lobata* Ohwi, and *L. japonicus* (CPL) on vascular endothelial dysfunction in ovariectomized rats. Specifically, we investigated the potential effects of CPL extracts on reducing inflammation, oxidative stress, and lipid accumulation, as well as promoting vasodilation and improving blood circulation. We also sought to elucidate the underlying mechanisms of these effects using ox-LDL-induced HUVECs.

## 2. Results

### 2.1. E-Screening of CPL Extracts Using MCF-7 Cells

**Figure 1:**
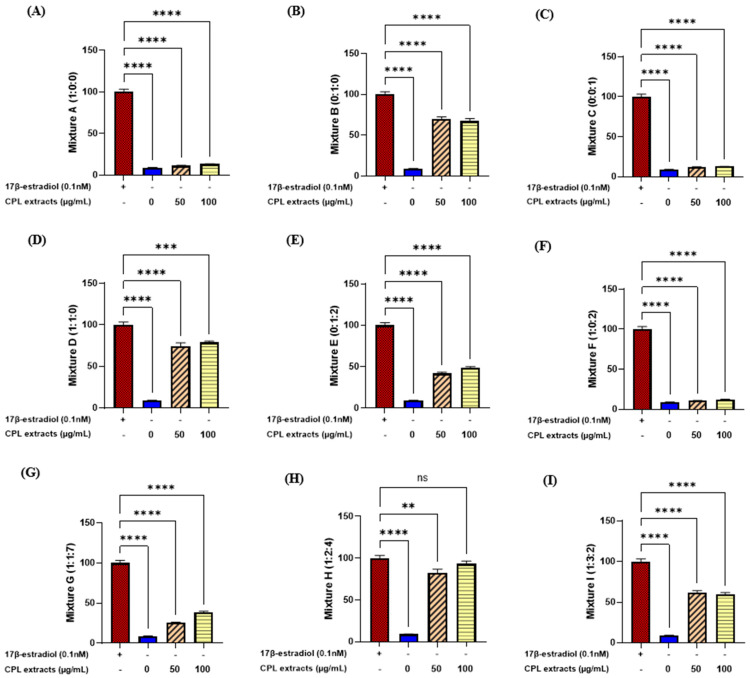
(**A**–**I**) Cell proliferation in MCF-7 cells at different ratios. MCF-7 cells were treated with different ratios of CPL extracts (50 and 100 μg/mL). Values are presented as the mean ± SD. **** *p* < 0.0001, *** *p* < 0.001, ** *p* < 0.01 vs. 17β-estradiol treatment; ns: non-significant.

We tested nine different types of mixtures, using MCF-7 cells to examine estrogen-like activity. Only mixture H (1:2:4) showed no significantly different activity in the CPL extract dose of 100 μg/mL compared with the positive control, 17β-estradiol (Figure 1). Therefore, the most effective mixture ratio of the CPL extracts was ascertained to be 1:2:4.

### 2.2. High-Performance Liquid Chromatography (HPLC) Analysis of CPL Extracts

**Figure 2:**
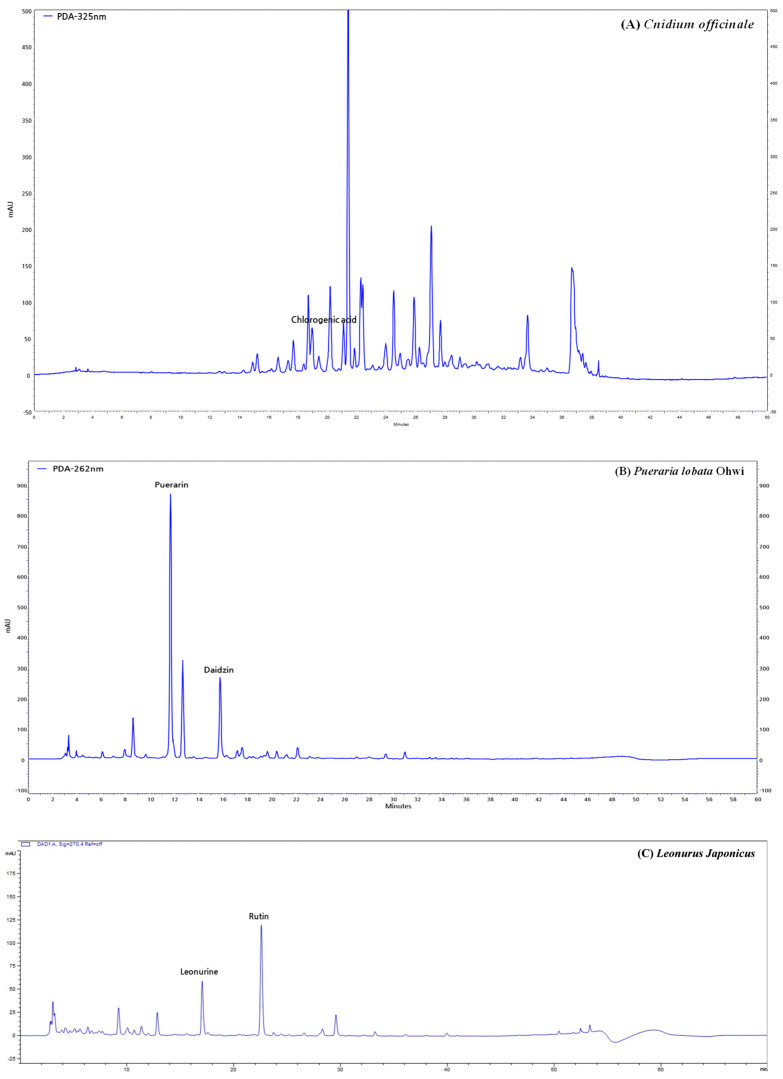
High-performance liquid chromatography (HPLC) analysis of CPL extracts. *C. officinale* (**A**), *P. lobata* Ohwi (**B**), *L. japonicus* (**C**).

We performed HPLC to identify the CPL extracts and the data of which are shown in Figure 2. HPLC profiles revealed chlorogenic acid in *C. officinale*; puerarin and daidzin in *P. lobata* Ohwi; and leonurine and rutin in *L. japonicus*.

### 2.3. Body Weight and Body Weight Gain

**Figure 3:**
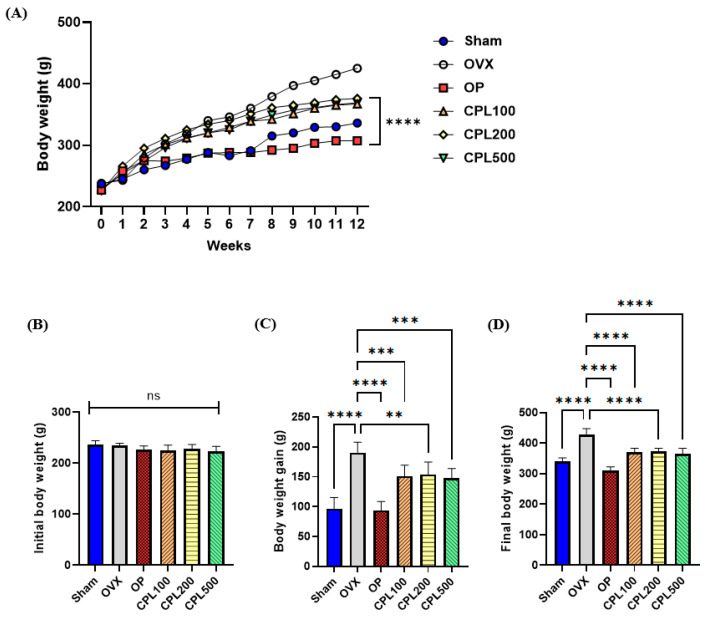
Body weight changes in ovariectomized rats. Body weight change (**A**), initial body weight (**B**), body weight gain (**C**), and final body weight (**D**). Sham: non-ovariectomized but sham-operated control; OVX: ovariectomized control; OP: ovariectomy + positive control (1 mg/kg b.w. of 17β-estradiol); CPL100: ovariectomy + CPL extracts (100 mg/kg b.w.); CPL200: ovariectomy + CPL extracts (200 mg/kg b.w.); CPL500: ovariectomy + CPL extracts (500 mg/kg b.w.). Values are presented as the mean ± SD. **** *p* < 0.0001, *** *p* < 0.001, ** *p* < 0.01 vs. OVX; ns: non-significant.

There were no significant variations in the starting body weight of any group (Figure 3A,B), but the ultimate body weight gains of all groups were significantly higher than their starting body weight (Figure 3C). The final body weight data of the OVX group revealed a noticeably larger increase compared with the other groups (Figure 3D). Following a 12-week treatment with CPL extracts, the ultimate body weight of the treatment groups was notably lower than that of the OVX group.

### 2.4. Organ and Fat Weight

**Figure 4:**
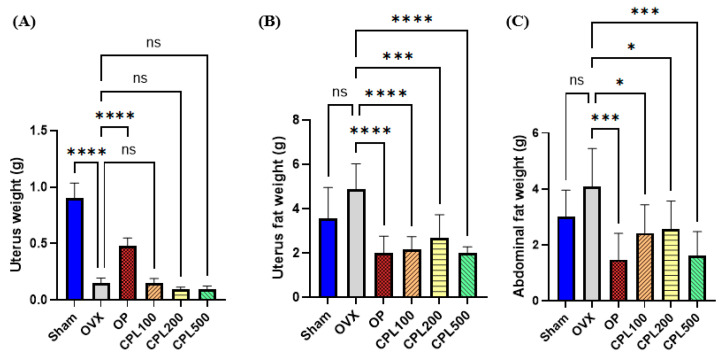
Organ and fat weights of ovariectomized rats. Uterus (**A**), uterus fat (**B**), and abdominal fat (**C**). Values are presented as the mean ± SD. **** *p* < 0.0001, *** *p* < 0.001, and * *p* < 0.05 vs. OVX; ns: non-significant.

The uterine weights of the Sham and OP groups were higher than those of the OVX and CPL-extract-treated groups (Figure 4A). There were no significant differences in uteri after 12 weeks of CPL extract administration compared with the OVX group (Figure 4A). OVX rats had higher abdominal and uterine fat weights than the OP group and the groups that received the CPL extracts (Figure 4B,C).

### 2.5. Tail Temperatures

**Figure 5:**
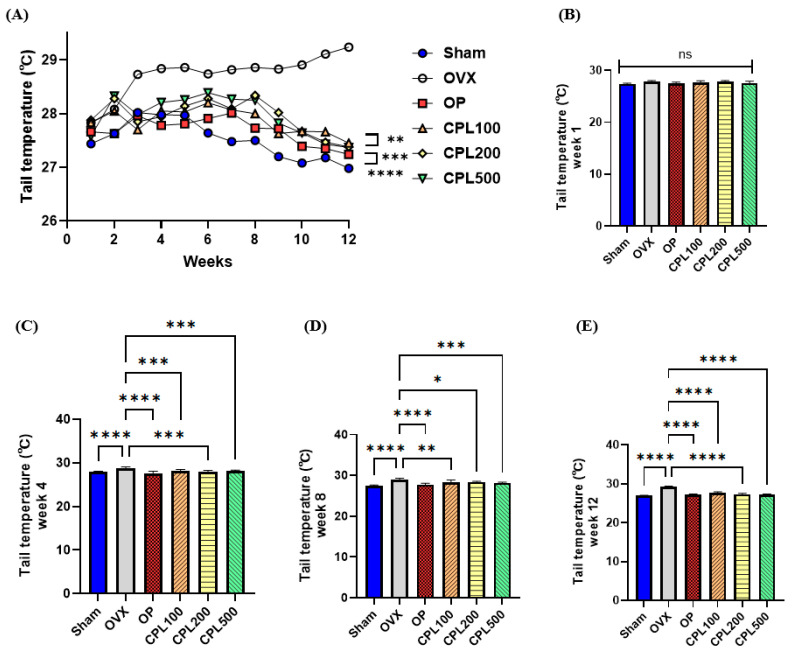
Tail temperature changes in ovariectomized rats. Tail temperature change (**A**), week 1 (**B**), week 4 (**C**), week 8 (**D**), and week 12 (**E**). Values are presented as the mean ± SD. **** *p* < 0.0001, *** *p* < 0.001, ** *p* < 0.01, and * *p* < 0.05 vs. OVX; ns: non-significant.

There were no significant variations in tail temperatures in the first week across all groups (Figure 5B). In week 4, the OVX tail temperature dramatically increased and was the highest among all groups (Figure 5C). In week 8, the OP group was administered 17β-estradiol and displayed lower temperatures than in week 4 (Figure 5C,D). The tail temperatures of the CPL-extract-treated groups were significantly lower than those of the OVX group. The OVX group displayed the highest tail temperatures among all groups in the previous week and week 12. The Sham, OP, CPL100, CPL200, and CPL500 groups displayed much lower temperatures than the OVX group (Figure 5E).

### 2.6. Serum Lipid Profiles

**Figure 6:**
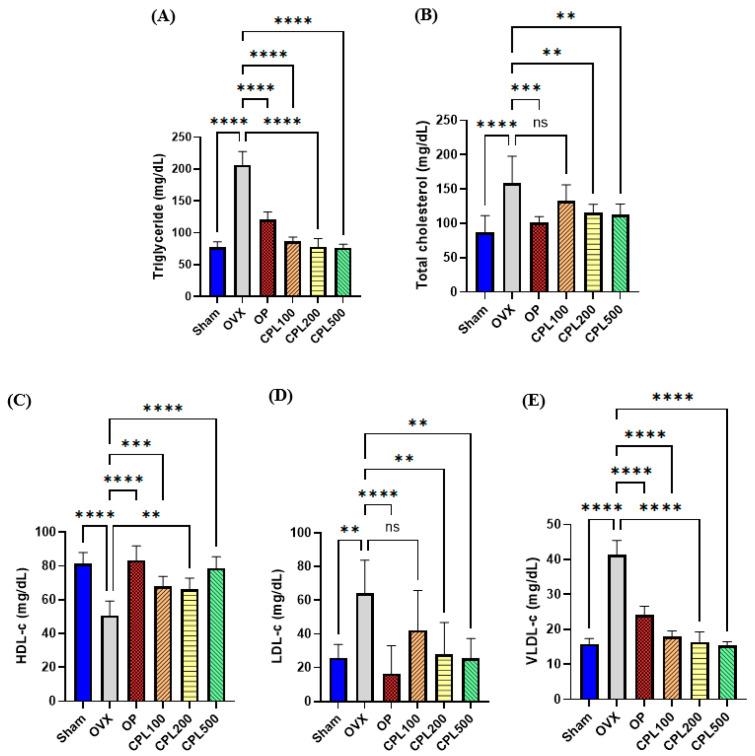
Serum lipid profiles of ovariectomized rats. TG (**A**), TC (**B**), HDL-C (**C**), LDL-C (**D**), and VLDL-C (**E**). Values are presented as the mean ± SD. **** *p* < 0.0001, *** *p* < 0.001, and ** *p* < 0.01 vs. OVX; ns: non-significant.

Following ovariectomy, the HDL-C levels decreased but the TG, TC, LDL-C, and VLDL-C levels increased. The OVX group showed the highest serum TG and VLDL-C levels among the ovariectomized groups (Figure 6A,E), whereas it had the lowest HDL-C levels (Figure 6C). The CPL extract groups (100, 200, and 500 mg/mL) showed considerably lower TG and VLDL-C levels than the OVX group following the 12-week administration of CPL extracts. The CPL200 and CPL500 groups also showed significantly decreased serum TC levels (Figure 6B); these levels were significantly lower than those of the OVX group (Figure 6D).

### 2.7. Serum Hepatic Function Profiles

**Figure 7:**
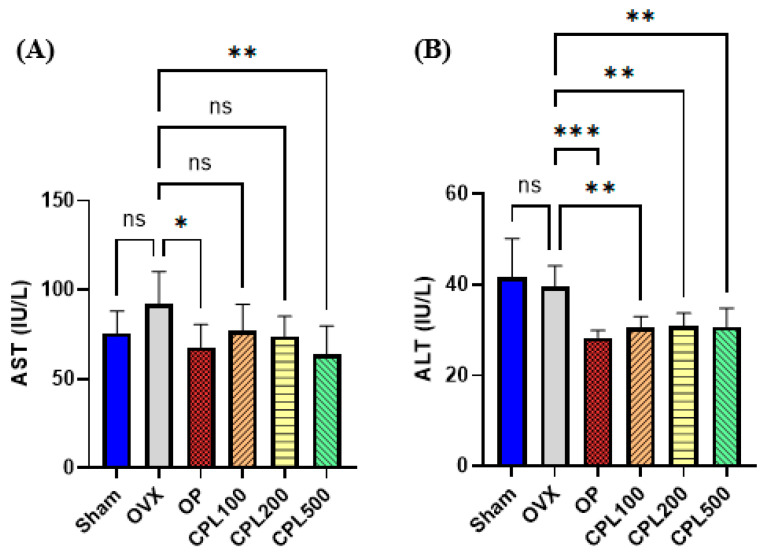
Serum hepatic function profiles of ovariectomized rats. AST (**A**) and ALT (**B**). Values are presented as the mean ± SD. *** *p* < 0.001, ** *p* < 0.01, and * *p* < 0.05 vs. OVX; ns: non-significant.

AST levels were significantly reduced in the CPL500 group compared with the OVX group following a 12-week treatment with CPL extracts. ALT levels were significantly reduced across all dose groups compared with the OVX group (Figure 7).

### 2.8. Serum Estradiol (E2) Levels

**Figure 8:**
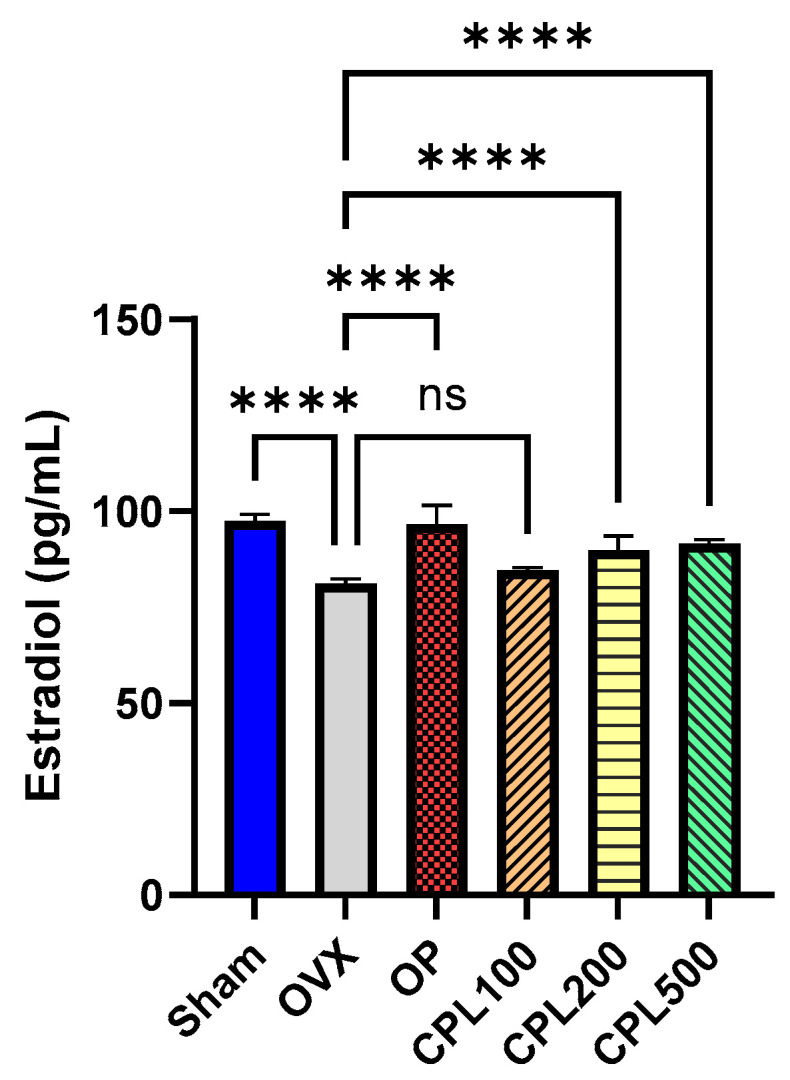
Serum estradiol (E2) levels of ovariectomized rats. Values are presented as the mean ± SD. **** *p* < 0.0001 vs. OVX; ns: non-significant.

The estrogen levels of the CPL100 group were not significantly different from those of the OVX group (Figure 8).

### 2.9. Serum Vasoactive Substances

**Figure 9:**
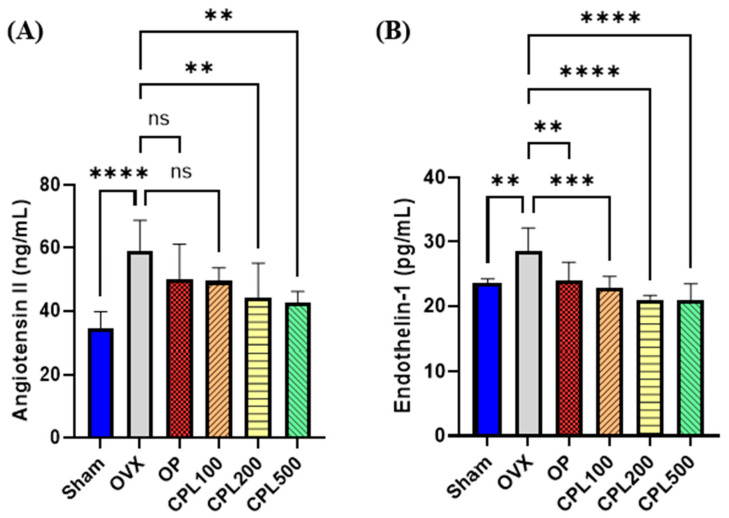
Serum vasoactive substances in ovariectomized rats. Ang-II (**A**) and ET-1 (**B**). Values are presented as the mean ± SD. **** *p* < 0.0001, *** *p* < 0.001, and ** *p* < 0.01 vs. OVX; ns: non-significant.

The OVX group had the highest angiotensin II (Ang-II) concentration among all groups (Figure 9A). Ang-II levels were notably lower after CPL extract administration at 200 and 500 mg/mL for 12 weeks compared with the OVX group. The OVX group had the highest endothelin 1 (ET-1) concentration among all groups (Figure 9B). Following a 12-week administration of 100, 200, or 500 mg/mL CPL extracts, the ET-1 concentration levels were observed to be considerably lower than those of the OVX group.

### 2.10. Serum Cardiovascular Risk Indices

**Figure 10:**
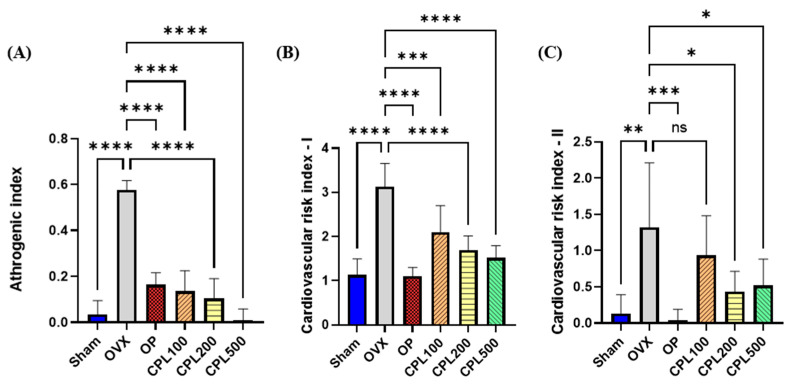
Serum cardiovascular risk indices of ovariectomized rats. AI (**A**), CRI-I (**B**), and CRI-II (**C**). Values are presented as the mean ± SD. **** *p* < 0.0001, *** *p* < 0.001, ** *p* < 0.01, and * *p* < 0.05 vs. OVX; ns: non-significant.

Our findings revealed that the atherogenic index (AI), cardiovascular risk index I (CRI-I), and cardiovascular risk index II (CRI-II) were all greatest in the OVX group (Figure 10A–C). After administering CPL extracts for 12 weeks, AI, CRI-I, and CRI-II showed a significant decrease in all indices compared with the OVX group, with the exception of CRI-II in the CPL100 group.

### 2.11. Endometrium Thickness

**Figure 11:**
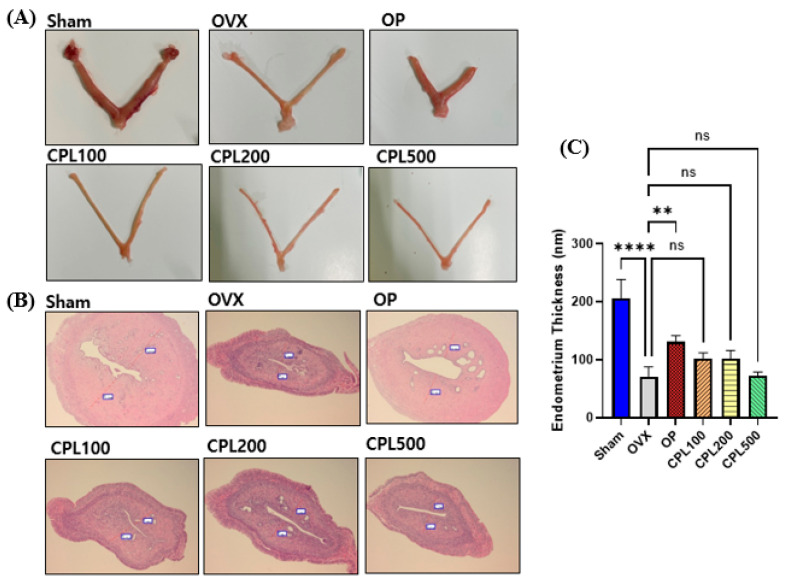
Representative images of the uterus and endometrium thickness in ovariectomized rats. Representative images (**A**), histological analyses of uteri (**B**), and endometrium thickness (**C**). Values are presented as the mean ± SD. **** *p* < 0.0001 and ** *p* < 0.01 vs. OVX; ns: non-significant.

Following a 12-week treatment regimen with CPL extracts, no significant changes were noted compared with the OVX group (Figure 11A–C).

### 2.12. Protein Expression Levels and Their Estrogenic Effect in the Uterus

**Figure 12:**
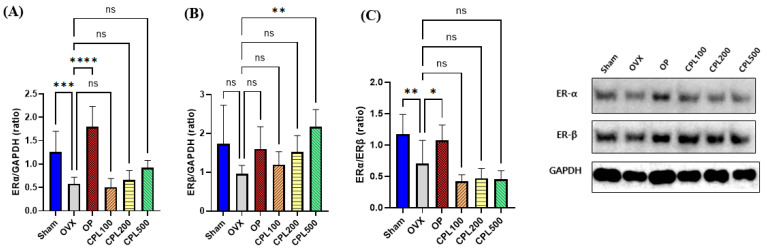
Protein expression of estrogen receptors in ovariectomized rats. ERα (**A**), ERβ (**B**), and ERα/ERβ (**C**). Values are presented as the mean ± SD. **** *p* < 0.0001, *** *p* < 0.001, ** *p* < 0.01, and * *p* < 0.05 vs. OVX; ns: non-significant.

The OVX group’s estrogen receptor α (ERα) protein expression was considerably lower following ovariectomy (Figure 12A). There were no discernible variations in ERα following the 12-week treatment with CPL extracts compared with the OVX group. There were no differences in the protein expression of ERβ between the Sham, OVX, OP, CPL100, and CPL200 groups (Figure 12B). However, the estrogen receptor β (ERβ) protein expression was considerably higher following 12 weeks of CPL extract administration at a dose of 500 mg/mL b.w. than in the OVX group. There were no discernible alterations in the Erα-to-ERβ ratio between the OVX group and the CPL extract treatment groups (Figure 12C).

### 2.13. Protein Expression Levels and Their Vasodilation Effect in the Liver

**Figure 13:**
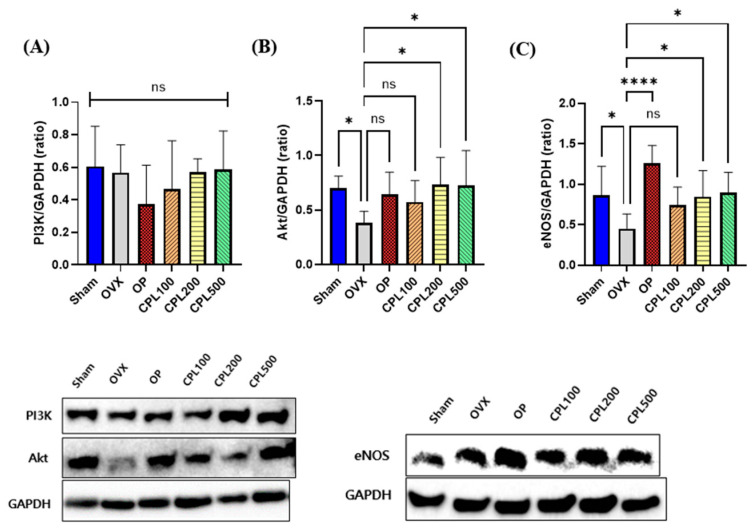
Protein expression levels of ovariectomized rats. PI3K (**A**), AKT (**B**), and eNOS (**C**). Values are presented as the mean ± SD. **** *p* < 0.0001 and * *p* < 0.05 vs. OVX; ns: non-significant.

The CPL500 group had the highest expression level across all groups for the PI3K protein, whereas the OP group had the lowest (Figure 13A). There were no appreciable variations in PI3K protein expression levels in any group. The OVX group had the lowest AKT protein expression level (Figure 13B). There was a considerable increase in the protein expression levels of AKT in the CPL200 and CPL500 groups following the 12-week CPL extract administration, which upregulated the eNOS expression. The OVX group had the lowest eNOS protein expression among all groups (Figure 13C); there were no appreciable changes in the Sham, CPL100, and CPL500 groups. The OP, CPL200, and CPL500 groups displayed much higher expression levels than the OVX group.

### 2.14. Cell Adhesion Molecule Levels in HUVECs

**Figure 14:**
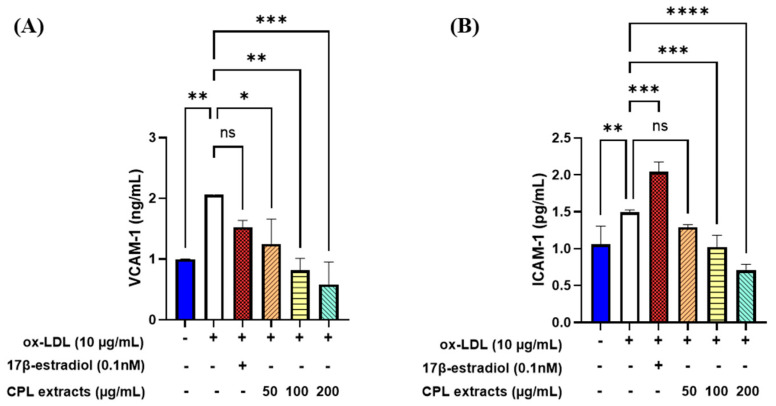
CPL extracts on endothelial activation in HUVECs. VCAM-1 (**A**) and ICAM-1 (**B**). HUVECs were induced with ox-LDL (10g/mL) and treated with CPL extracts (0, 50, 100, and 200 μg/mL). Values are presented as the mean ± SD. **** *p* < 0.0001, *** *p* < 0.001, ** *p* < 0.01, and * *p* < 0.05 vs. 17β-estradiol treatment; ns: non-significant.

The VCAM-1 levels significantly decreased after CPL extract treatments of 50, 100, and 200 μg/mL (Figure 14). The ICAM-1 levels did not significantly decrease after the CPL extract treatment of 50 μg/mL but they significantly decreased in the 100 and 200 μg/mL treatments compared with the ox-LDL treatment.

### 2.15. Gene Expression Levels in HUVECs

**Figure 15:**
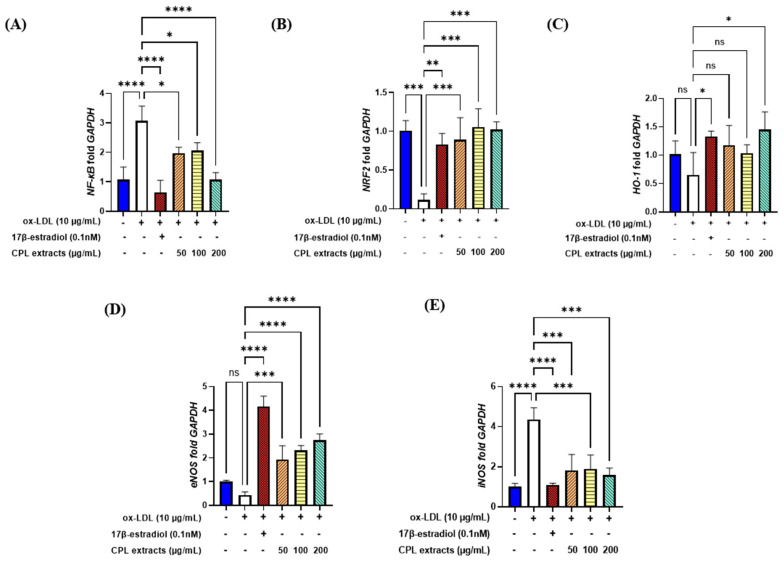
Gene expression levels of CPL extracts in HUVECs. *NF-κB* (**A**), *NRF2* (**B**), *HO-1* (**C**), *eNOS* (**D**), and *iNOS* (**E**). Values are presented as the mean ± SD. **** *p* < 0.0001, *** *p* < 0.001, ** *p* < 0.01, and * *p* < 0.05 vs. 17β-estradiol treatment; ns: non-significant.

Our results indicated that ox-LDL induction led to the highest levels of *NF-κB*, the lowest levels of *NRF2*, and decreased levels of *HO-1*. After treatment (50, 100, and 200 μg/mL), the CPL extracts significantly decreased *NF-κB* and increased the level of *NRF2* at all doses. *HO-1* increased; it significantly increased in the 200 μg/mL CPL extract group (Figure 15A–C).

*eNOS* significantly increased and *iNOS* significantly decreased after treatment with CPL extracts compared with the ox-LDL-induced control (Figure 15D,E).

## 3. Discussion

The aim of this study was to evaluate the cardioprotective effects of mixtures of CPL extracts on vascular endothelial dysfunction by reducing inflammation, oxidative stress, and lipid accumulation, as well as promoting vasodilation to improve blood circulation in ovariectomized rats. We also aimed to elucidate the mechanisms underlying these effects in ox-LDL-induced HUVECs. Overall, we observed that these effects were linked to the antioxidant, anti-inflammatory, and cardioprotective properties of the CPL extracts.

We examined the serum AST and ALT levels to assess the toxicity of the medication, as they are thought to be serum indicators of hepatic function and elevated levels are indicative of liver damage [12]. We measured the levels of these hepatic indicators of liver function to assess the effects of CPL extracts in different rat groups. The results demonstrated that menopause affects AST and ALT levels, and that CPL extracts could reduce hepatic indicators by correcting aberrant amounts of these enzymes.

Concerns regarding the safety of estrogen during menopausal treatment are widespread. Elevated estrogen levels beyond the recommended range and their impact on the uterus can increase the risk of breast cancer and endometrial cancer [13]. The results for estrogenic effects demonstrated that estradiol levels could be affected by menopause and that CPL extracts did not result in any levels over the reference range for hormones, which is between 71 and 128 pg/mL [14]. The endometrium is the main target tissue of estrogen [15]. Ovariectomy modifies uterine layers and decreases the cell count and endometrial epithelial thickness, as evidenced by earlier research [16]. Estrogen receptor α (ERα) and estrogen receptor β (ERβ) are the two main estrogen receptors. Through binding to specific DNA regulatory regions, these proteins regulate the activity of estrogenic substances. The uterus, the ovary, and adipose tissue are among the organs where ERα is primarily expressed. ERβ is primarily expressed in the immune system, epithelium, and ovaries [17]. In this study, we assessed the protein expression of ERβ and ERβ in the uteri of ovariectomized rats treated with CPL extracts. We observed that ERβ was upregulated. This helps to shield the uterus from the hazards caused by estrogen.

Increases in body and fat weight may be related to an imbalance in energy metabolism caused by estrogen deficiency [18]. We observed that 12 weeks of treatment with CPL extracts lowered body weight, body weight gain, uterine fat weight, and abdominal fat weight in ovariectomized rats. An imbalance in the TG, TC, LDL-C, and HDL-C levels is known as dyslipidemia [18]. The lipid profiles in this study revealed that the OVX group had higher TG, TC, LDL-C, and VLDL-C levels. However, OVX-induced alterations in the TG, LDL-C, and VLDL-C lipid profiles were dramatically decreased and HDL-C levels were significantly elevated after treatment with CPL extracts, demonstrating the advantageous effects of CPL extracts.

Both menopause and aging increase the risk of cardiovascular disorders [19]. There was a significant decrease in all cardiovascular risk indices, including AI, CRI-II, and CRI-II, following a 12-week treatment with CPL extracts compared with the OVX group. Ang-II and ET-1 are important vasoconstrictor proteins in the vascular system that enhance oxidative stress by inducing inflammation and increasing the levels of ROS [20]. There was a decrease in serum vasoactive chemicals following a 12-week treatment with CPL extracts, indicating that CPL extracts could support the vascular system’s antioxidative and protective effects.

Hot flashes cause peripheral vasodilation, which is characterized by elevated skin temperatures and blood flow [21]. The capabilities of preventing oxidative stress, such as free-radical scavenging and neutralizing excess ROS, are reduced as a result of the decline in estrogen during menopause, which also affects lipid profiles and oxidative stress [22]. We observed the tail temperatures of each group for 12 weeks after injections to determine how CPL extracts affected hot flush relief in vivo. We observed that CPL extracts decreased tail temperature levels after 3 weeks of dosing.

Studies have indicated that the downstream serine/threonine protein kinase of the PI3K family, which constitutes the PI3K and AKT signaling pathways, plays a critical role in promoting cell proliferation and inhibiting apoptosis [23]. eNOS is necessary to control blood pressure and generate NO for vasodilation, helping to maintain and regulate a healthy cardiovascular system. AKT is phosphorylated via the PI3K signaling pathway, which activates eNOS and generates NO from phosphorylated AKT. The phosphorylation of the eNOS protein at serine 1179, which is connected to eNOS activity, is regulated by the PI3K/AKT signaling pathway [24,25,26]. The liver protein expression levels revealed that PI3K was upregulated (but not significantly), AKT was upregulated, and eNOS was activated in the CPL-extract-treated groups.

Aging and menopause are associated with increased oxidative stress and excessive generation of ROS, both of which are detrimental to the liver and cardiovascular systems [12]. Through their antioxidative and cardioprotective effects on blood pressure, cardiovascular disease, and blood circulation, CPL extracts can reduce menopause- and age-driven oxidative stress and inflammation [6,7,8,9,10,11]. In this study, we observed that the application of CPL extracts to ox-LDL-induced HUVECs resulted in decreased oxidative stress and inflammation levels. VCAM-1 and ICAM-1 are cell adhesion molecules (CAMs) that mediate macrophage adherence and infiltration [27]. The administration of CPL extracts led to a dose-dependent decrease in VCAM-1 and ICAM-1 levels. *HO-1* is an enzyme induced by *NRF2*, which is crucial for antioxidant defense against various inflammatory and oxidative stressors. *NF-kB* is stimulated by increased ROS in endothelial cells, which are proinflammatory markers [28]. *NRF2* and numerous antioxidant genes are transcribed by a redox-sensitive transcription factor [29]. We observed that *NF-kB* gene expression was downregulated following treatment with CPL extracts. NRF2 and HO-1 produce positive effects by protecting endothelial cells from oxidative damage [30]. The activation of *NRF2*, which encourages *HO-1* expression, inhibits oxidative stress and increases NO and *eNOS* production. *eNOS*, a vasodilation component, decreases with age and menopause, increasing the risk of cardiovascular diseases [31]. The inflammatory marker *iNOS* causes excessive NO production, which can lead to atherosclerosis. *eNOS* is involved in vasodilation, which reduces after menopause and aging, thereby increasing the risk of cardiovascular illnesses. Our research demonstrated that CPL extracts could protect the vascular system by upregulating *eNOS* in endothelial cells during vasodilation and downregulating *iNOS* during inflammatory conditions.

## 4. Materials and Methods

### 4.1. Preparation of CPL Extracts

CPL extracts were provided by Kwangdong Pharmaceutical Co., Ltd. (Seoul, Republic of Korea). The product ratios of each raw material are listed in Table 1. In brief, the materials (which were recombined by applying the extraction yield of individual substance; *C. officinale* (*Cnidii rhizoma*): *P. lobata* (*Puerariae radix*): *L. japonicus* (*Leonuri herba*), 1:2:4 were extracted in 30%(*v*/*v*) ethanol for 8 h, filtered, evaporated, and spray dried. The dry residue was stored at −80 °C.

### 4.2. Animals

Forty-two 9-week-old female Sprague Dawley rats (Saeronbio Inc., Uiwang, Republic of Korea), weighing 200–220 g, were kept in a controlled environment with a 12 h light/dark cycle at temperature of 22 ± 1 °C and humidity of 50–60%. They also had unrestricted access to water and the Teklad Global 18% Protein Rodent Diet (Teklad Diets, Madison, WI, USA). The animal study protocols were approved on 19 April 2021 by the Institutional Animal Care and Use Committee (IACUC) of Sookmyung Women’s University for the Care and Use of Laboratory Animals (SMWU-IACUC-2104-004).

### 4.3. Ovariectomy Procedure and Treatment

After a week of acclimatization, 7 rats underwent sham surgery and 35 underwent bilateral ovariectomy. The rats were then randomized into five groups following a 2-week recuperation period, with seven individuals in each of the following groups: Sham (non-ovariectomized but sham-operated control); OVX (ovariectomized control); OP (ovariectomy + positive control (1 mg/kg b.w. of 17β-estradiol) (Sigma Aldrich, St. Louis, MO, USA)); CPL100 (ovariectomy + CPL extracts (100 mg/kg b.w.)); CPL200 (ovariectomy + CPL extracts (200 mg/kg b.w.)); and CPL500 (ovariectomy + CPL extracts (500 mg/kg b.w.)). The rats were orally administered each treatment for 12 weeks.

### 4.4. Body Weight Measurement

Body weights were measured weekly. The body weight gain was determined by subtracting the final weight from the initial weight.

### 4.5. Serum Biochemical Analysis

Total cholesterol (T-CHO) (3I2020; Asanpharm, Hwaseong, Republic of Korea), triglyceride (TG-S) (3I1570; Asanpharm), and HDL cholesterol levels (HDL-CHO) (3I2030; Asanpharm) were measured using an Epoch microplate spectrophotometer (BIOTEK, Inc., Winooski, VT, USA). Serum LDL cholesterol and very-low-density lipoprotein (VLDL) cholesterol levels were calculated using the formulas mentioned in a previous study [32].

Serum estradiol (E2) levels were measured using an E2 ELISA kit (E-OSEL-R0001; Elabscience Biotechnology Co., Ltd., Wuhan, China). Aspartate aminotransferase (AST) and alanine aminotransferase (ALT) levels were measured using assay kits (AM103-K and AM102; Asanpharm, Seoul, Republic of Korea). The serum concentration of angiotensin II (Ang-II) was measured using an Ang-II ELISA kit (E-EL-R1430; Elabscience), and endothelin 1 (ET-1) in serum was measured using an ET-1 ELISA kit (E-EL-R1458; Elabscience).

### 4.6. Cardiovascular Index

The atherogenic index (AI) and cardiovascular risk index (CRI) I and CRI-II were calculated using the Haglund method [33].

### 4.7. Tail Temperature Measurements

An infrared thermometer (153-IRB; BiosebLab, Paris, France) was used twice a week to measure the temperature of tails 2 cm away from the rectum.

### 4.8. Uterine Histological Analysis

The samples were fixed in a 10% neutral buffered formalin solution (Sigma-Aldrich, St. Louis, MO, USA) to quantify the endometrial length of the uteri. Fixed tissues were sectioned at a thickness of 5 µm and stained with hematoxylin and eosin (H&E) for a pathological analysis to measure the length of the uteri. The endometrial thickness was measured at 100× magnification.

### 4.9. Western Blotting

Subsequently, 0.01 g ± 0.002 g of the tissues was homogenized with PRO-PREP Protein Extraction Solution (17081; iNtRON, Biotechnology, Seongnam, Republic of Korea). The protein concentration was measured using a PRO-MEASURE ™ kit (21011; iNtRON). The primary antibodies obtained after an entire night at 4 °C were blocked for 1 h in a blocking buffer and were as follows: ERα (1:1000; SC-8005; Santa Cruz Biotechnology, Inc., Dallas, TX, USA), ERβ (1:1000; SC-53494; Santa Cruz, Dallas, TX, USA), PI3K (1:1000; 4292S; Cell signaling Technology (CST), Danvers, MA, USA), AKT (1:1000; 9272S; CST, Danvers, MA, USA), eNOS (1:1000; 92027S; CST, Danvers, MA, USA), and GAPDH (1:5000; 5174S; GeneTex, Inc., Irvine, CA, USA). The membrane was incubated for 2 h with anti-rabbit IgG and horseradish peroxidase (HRP)-linked antibodies (1:3000; 7074; CST, Danvers, MA, USA). Chemiluminescence detection was analyzed via a densitometric analysis (Biomolecular Imaging System-3; Ge Healthcare, Chicago, IL, USA) at the Chronic and Metabolic Diseases Research Center of Sookmyung Women’s University.

### 4.10. Cell Proliferation Assay

MCF-7 cells were purchased from the Korea cell line bank (KCLB; Seoul, Republic of Korea) and cultured following the process of a previous study [11]. The proliferation of MCF-7 cells (Table 1) was assessed after 7 days of growth. Each well was filled with 100 µL thiazolyl blue tetrazolium bromide (Sigma-Aldrich); this solution had a concentration of 5 mg/mL. The cells were then incubated for 4 h at 37 °C in 5% CO_2_. A total of 1 mL of dimethyl sulfoxide (DMSO; Sigma-Aldrich) was used instead of the medium. The absorbance was then measured [34].

### 4.11. Soluble Vascular Cell Adhesion Molecule-1 and Soluble Intercellular Adhesion Molecule-1 Assay (sVCAM-1 and sICAM-1 Assay)

HUVECs were purchased from the American Type Culture Collection (ATCC) and followed a previous study [35,36]. The cells were treated with 17β-estradiol (0.1 nM) (E4389; Sigma-Aldrich) for the positive control and different concentrations of CPL extracts (0, 50, 100, and 200 µg/mL) in each different well. After 2 h incubation, the cells were induced with ox-LDL (10 µg/mL) (10 µM CuSO4) for 40 h for experimental use. VCAM-1 was measured using a VCAM-1 Simple Step ELISA kit (ab223591; Abcam, Cambridge, UK) and ICAM-1 was measured using an ICAM-1 (CD54) Human SimpleStep ELISA kit (ab174445; Abcam) following the manufacturer’s instructions.

### 4.12. mRNA Expression Analysis

HUVECs were seeded in 6-well culture plates at a density of 1 × 10^6^ cells/cm^2^. Subsequently, either 17β-estradiol (0.1 nM) was used as a positive control in each treatment or various CPL extract doses (0, 50, 100, and 200 µg/mL) were used for 24 h [35,36,37]. Thereafter, the cells were induced with ox-LDL (10 µg/mL) (10 µM CuSO4) for 4 h for experimental use. RNAiso Plus (9108; Takara Bio Inc., Shiga, Japan) was used to extract RNA. The RNA purity was determined using an absorbance ratio of 260/280 nm. PrimeScript RT Master Mix (Perfect Real Time) (RR360A; Takara, Shiga, Japan) was used for cDNA synthesis. TB Green Premix was used as the PCR product. The PCR products were analyzed using a LightCycler 96 (Roche Molecular Systems, Inc., Basel, Switzerland) after being combined with TB Green Premix Ex TaqTM (Tli RNaseH Plus) (RR420A; Takara). Each datapoint was standardized to *GAPDH* to calculate the fold change for the relative quantitative analysis.

### 4.13. Statistical Analysis

Statistical analyses were performed using the Prism 10.131 software program (GraphPad Software Inc., San Diego, CA, USA). The results for each group were analyzed using a one-way analysis of variance followed by Tukey’s multiple comparison test. Significance was defined as follows: **** *p* value < 0.0001, *** *p* < 0.001, ** *p* < 0.01, and * *p* < 0.05.

## 5. Conclusions

The findings of our study suggest that CPL extracts could improve vascular health by enhancing adhesion molecules, reducing atherogenic indices, promoting vasodilation, and improving blood circulation, ultimately alleviating vasomotor symptoms and endothelial dysfunction. Although numerous studies have explored the effects of traditional Chinese medicines on menopause and cardiovascular health, our research is the first to specifically investigate the cardioprotective effects of CPL extracts in ovariectomized rats as well as the associated molecular mechanisms in HUVECs. Further clinical trials are necessary to confirm the effectiveness and safety of CPL extracts in postmenopausal women. Future research should explore the long-term effects of CPL extracts and their potential for use in combination with other therapeutic approaches to prevent cardiovascular diseases in menopausal populations.

## Figures and Tables

**Table 1:** Mixing ratio of raw materials of *C. officinale*, *P. lobata* Ohwi, and *L. japonicus*.

Mixture	*Cnidii rhizoma*	*Puerariae radix*	*Leonuri herba*
A	1	0	0
B	0	1	0
C	0	0	1
D	1	1	0
E	0	1	2
F	1	0	2
G	1	1	7
H	1	2	4
I	1	3	2

## Data Availability

The data presented in this study are available on request from the corresponding author. The data are not publicly available due to privacy.
